# Myelin-water imaging and multi-shell diffusion-weighted imaging in adults with adrenoleukodystrophy

**DOI:** 10.1093/braincomms/fcaf371

**Published:** 2025-09-25

**Authors:** Hemmo A F Yska, Menno D Stellingwerff, Stefan D Roosendaal, Marije M C Voermans, Marjo S van der Knaap, Petra J W Pouwels, Marc Engelen

**Affiliations:** Department of Child Neurology, Amsterdam Leukodystrophy Center, Emma Children's Hospital, Amsterdam University Medical Center, Amsterdam, Netherlands; Amsterdam Neuroscience, University of Amsterdam, Amsterdam, Netherlands; Department of Child Neurology, Amsterdam Leukodystrophy Center, Emma Children's Hospital, Amsterdam University Medical Center, Amsterdam, Netherlands; Amsterdam Neuroscience, VU University, Amsterdam, Netherlands; Department of Radiology and Nuclear Medicine, Amsterdam University Medical Center, Amsterdam, Netherlands; Department of Radiology and Nuclear Medicine, Amsterdam University Medical Center, Amsterdam, Netherlands; Department of Child Neurology, Amsterdam Leukodystrophy Center, Emma Children's Hospital, Amsterdam University Medical Center, Amsterdam, Netherlands; Amsterdam Neuroscience, University of Amsterdam, Amsterdam, Netherlands; Department of Child Neurology, Amsterdam Leukodystrophy Center, Emma Children's Hospital, Amsterdam University Medical Center, Amsterdam, Netherlands; Amsterdam Neuroscience, VU University, Amsterdam, Netherlands; Department of Integrative Neurophysiology, Center for Neurogenomics and Cognitive Research, Vrije Universiteit Amsterdam, Amsterdam, Netherlands; Department of Radiology and Nuclear Medicine, Amsterdam University Medical Center, Amsterdam, Netherlands; Department of Child Neurology, Amsterdam Leukodystrophy Center, Emma Children's Hospital, Amsterdam University Medical Center, Amsterdam, Netherlands; Amsterdam Neuroscience, University of Amsterdam, Amsterdam, Netherlands

**Keywords:** leukodystrophy, translational research, biomarker, radiology, imaging

## Abstract

The pathophysiology of X-linked adrenoleukodystrophy (ALD) is not well-understood. New quantitative MRI (qMRI) sequences, such as myelin-water imaging (MWI) and multi-shell diffusion-weighted imaging (DWI), are non-invasive techniques that can investigate microstructural abnormalities in the brain. Using these techniques, this study investigated abnormal white matter of cerebral ALD (cALD) lesions and normal-appearing white matter (NAWM) in ALD and in controls. Adult participants were scanned on 3T MRI scanners. Fractional anisotropy (FA), axial diffusivity (AD) and radial diffusivity (RD) were measured with diffusion tensor imaging (DTI). The neurite density index (NDI) was established using neurite orientation dispersion and density imaging (NODDI). Myelin water fractions (MWF) were evaluated with multi-compartment relaxometry diffusion-informed MWI (MCR-DIMWI) and multi-echo T2-relaxation imaging with compressed sensing (METRICS). Measures were evaluated in 10 regions of interest (ROIs) in participants with and without cALD, and in controls. A total of 81 ALD participants (69% males, of whom 17 had cALD), and 21 controls were included. FA, NDI, MWF_METRICS_ and MWF_MCR-DIMWI_ were significantly lower in lesions in the corpus callosum of cALD participants than in controls. FA, NDI and MWF_METRICS_ also significantly differentiated the NAWM of male, but not female, ALD participants from controls. FA and NDI had the largest effect sizes for males. In the NAWM, RD was increased, whereas AD was mostly unaffected. In a few NAWM regions, qMRI measures also differed between males with and without cALD. The NAWM of males with ALD contains microstructural abnormalities. Myelin and myelinated axons are impacted by ALD pathophysiology. These techniques have potential for clinical applications.

## Teaser

Yska *et al*. show that the new quantitative MRI techniques, myelin-water imaging and multi-shell diffusion-weighted imaging, are sensitive to (early) microstructural changes in the brains of patients with the metabolic disease adrenoleukodystrophy (ALD). These results provide clues to the pathophysiology of ALD and can have clinical value.

## Graphical Abstract

**Graphical Abstract:**
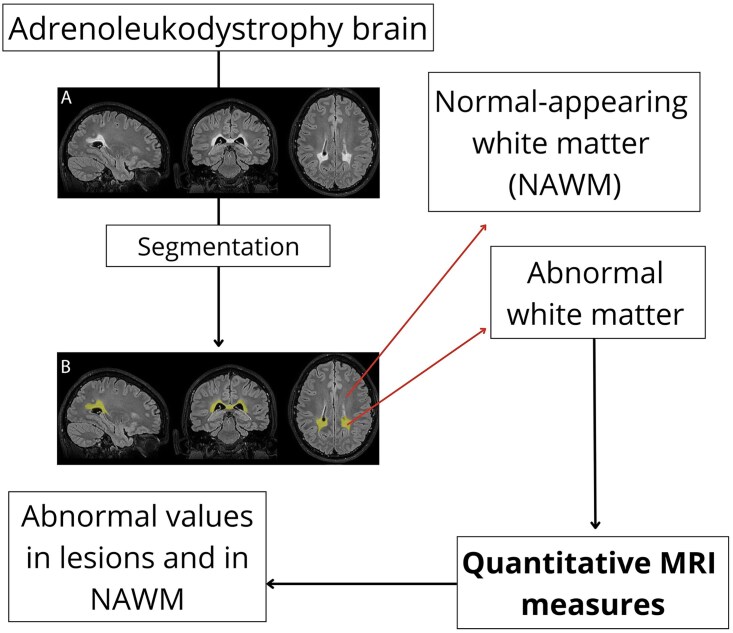

## Introduction

X-linked adrenoleukodystrophy (ALD) is a genetic neurometabolic disease characterized by increased very-long chain fatty acid (VLCFA; ≥C22) levels.^1^ People with ALD can develop a leukodystrophy [cerebral ALD (cALD)] with a highly specific pattern on MRI of the brain,^2^ and/or a slowly progressive myelopathy and peripheral neuropathy that primarily involves the corticospinal tracts and the dorsal columns.^3^ It is unclear why specific tissues are affected and why variability in disease manifestations is observed.^4,5^ Despite numerous studies, pathophysiology remains incompletely understood. The leukodystrophy of ALD is classified as affecting the myelin primarily,^6^ but it has been proposed that axonal degeneration actually precedes myelin degeneration.^7^ The incomplete understanding of ALD pathology is partly due to the fact that model systems (like the *ABCD1* knock-out mouse) do not recapitulate the disease well.^8^ Furthermore, histologic studies of post-mortem tissues represent end-stage disease and are therefore difficult to interpret as to which cell types are initially involved.

Magnetic resonance imaging (MRI) offers the advantage that changes in the brain can be observed *in vivo* and has provided important insights into disease progression in ALD.^9^ Conventional structural MRI techniques, however, lack the ability to detect subtle microstructural tissue abnormalities. Studies using quantitative MRI (qMRI) reported abnormalities in the brains of ALD patients without cALD and may be able to provide information on tissue microstructure.^10-16^ Two new qMRI techniques, myelin-water imaging (MWI) and neurite orientation dispersion and density imaging (NODDI), are especially promising in the context of ALD.^17^ MWI estimates the myelin-water fraction (MWF) in white matter^18-20^ and has been correlated to histological myelin stainings in post-mortem tissues in controls and in multiple sclerosis.^21,22^ NODDI is based on multi-shell diffusion-weighted imaging (DWI),^23^ which can also be used to generate the four single-shell diffusion tensor imaging (DTI) measures: fractional anisotropy (FA), mean diffusivity (MD), radial diffusivity (RD) and axial diffusivity (AD). RD is considered to reflect myelin, whereas AD better reflects axonal structure.^24,25^ White matter abnormalities in ALD have in the past been associated with decreased FA and increased RD in the brain.^13,26^ Similar results were found for DTI measures in the spinal cord.^27,28^ NODDI generates the neurite density index (NDI), orientation dispersion index and free isotropic water fraction. NDI was previously found to be most relevant in leukodystrophies^29^ and correlated with myelinated neurite density in rodent models.^30^ We hypothesize that microstructural abnormalities in ALD may be associated with a decreased NDI.

Abnormalities in qMRI measures could indicate onset of cALD before lesions become visible on conventional MRI. The primary aim of this study is to investigate abnormalities in MWI and multi-shell DWI in white matter lesions in a cross-sectional cohort of patients with cALD. The second aim is to investigate whether abnormalities can also be detected in the NAWM of people with cALD and in the NAWM of people without cALD, and whether these measures can help understand the pathophysiology of ALD.

## Materials and methods

### Participants and procedures

IRB approval was obtained at Amsterdam University Medical Center (# 2018_310) for a prospective natural history study (the Dutch ALD cohort). All participants provided informed consent prior to participation.^31^ MRI scans obtained during the ongoing cohort study from adults with ALD (male and female) were included. T2-weighted scans were evaluated for the presence of cALD by an experienced neuroradiologist.^2^ Twenty-two healthy controls of different ages and sexes were also included. Participants with widespread white matter abnormalities not related to ALD were excluded. This study adheres to the Strobe checklist for cohort studies.

### MRI acquisition

Participants were scanned on one of three 3-Tesla MR scanners (Ingenia Elition X or Ingenia, Philips Medical Systems, Best, Netherlands) using a 32-channel head coil. The protocol has been described in detail,^29^ with a scanning time of ∼ 40 min. Three conventional structural imaging sequences were acquired: 3D T1-weighted and 3D fluid attenuated inversion recovery (FLAIR), both at 0.9–1.0 mm isotropic resolution, and a 2D axial T2-weighted. All three qMRI sequences were acquired at 2.5 mm isotropic resolution. These included one multi-shell DWI sequence (with *b*-values 0, 1000 and 2000 s/mm^2^) and two MWI sequences: multi-echo gradient echo with variable flip angles (MGRE-VFA) and multi-echo T2 relaxation imaging with compressed sensing (METRICS).^18,19^ A B1-DREAM sequence was performed to correct for inhomogeneities of flip angles in the MGRE sequence. The combined use of multi-shell DWI and MGRE-VFA allowed for the calculation of multi-compartment relaxometry diffusion-informed MWI (MCR-DIMWI) and the associated MWF_MCR-DIMWI_.

### Segmentation

**Figure 1:**
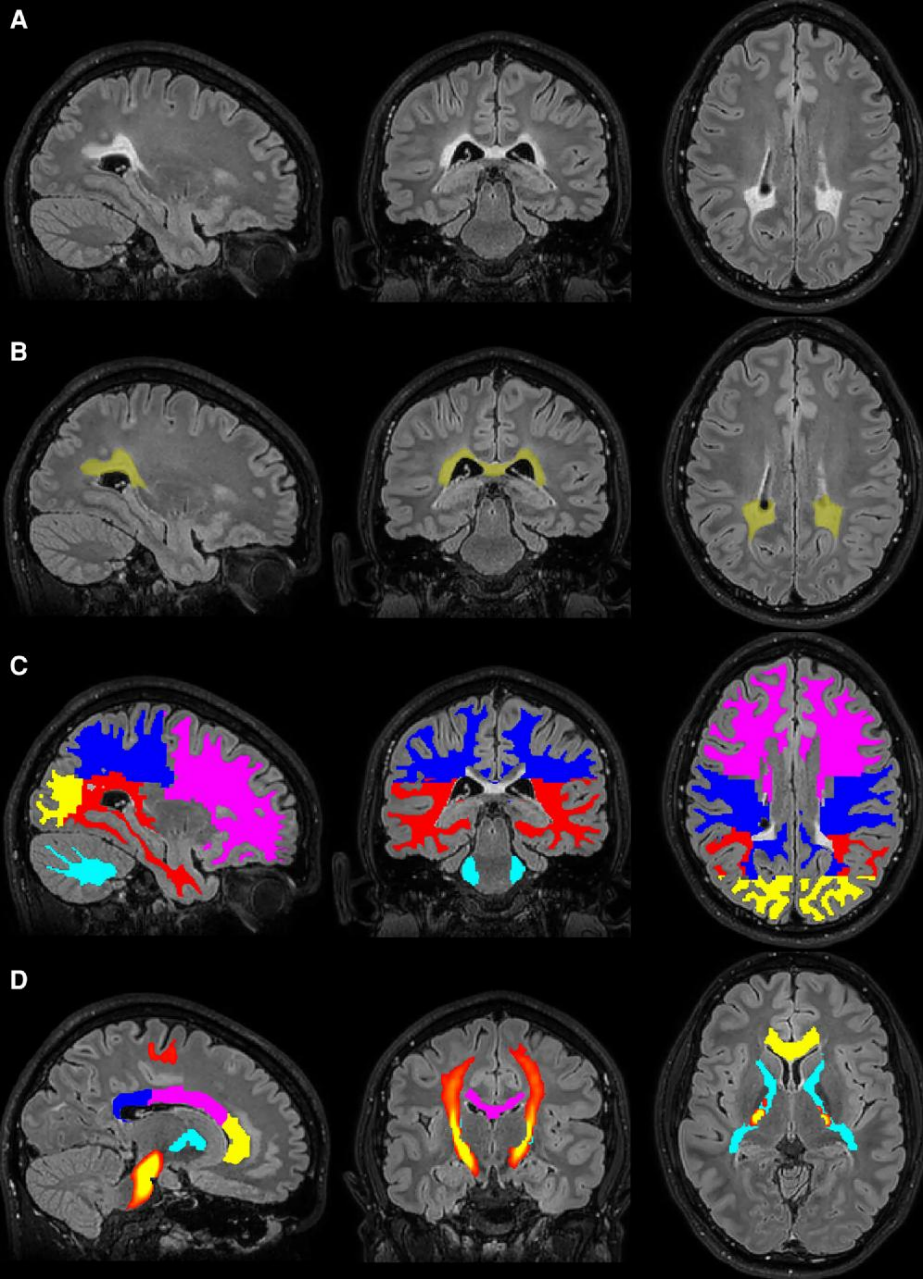
**Anatomical and lesion segmentation. (A**) Sagittal, coronal (posterior) and axial (centrum semiovale) images of a male with cALD. (**B**) Automatic lesion segmentation shown with yellow overlays. (**C**) Segmentation of the white matter of the cerebral lobes and of the cerebellum. Purple: frontal lobe; dark-blue: parietal lobe; yellow: occipital lobe; red: temporal lobe; light-blue: cerebellar white matter. (**D**) Yellow: genu of CC; purple: body of CC; dark-blue: splenium of CC; light-blue: internal capsule. Probabilistic tract segmentation of the corticospinal tracts is shown in red-yellow colours where yellow indicates a more certain localization. cALD, cerebral ALD; CC, corpus callosum.

Segmentation was performed according to the pipeline described by Stellingwerff *et al*.^29^ In short, automatic brain segmentation of 3D T1 was performed using Synthseg 2.0,^32^ and visually inspected. In addition, we performed automatic lesion segmentation of 3D T1 and 3D FLAIR using Lesion Segmentation Toolbox-AI (LST-AI), a deep learning method for lesion segmentation.^33^ Figure 1 shows an example of the segmentation results. DTI toolkit version 2.3.1 was used to register DWI brains to a common space, allowing registration of atlas-based ROIs.^34^ Using ANTs (Advanced Normalization Tools version 2.3.5), selected ROIs were registered to the MGRE-VFA, METRICS and multi-shell DWI data.^35^ We distinguished white matter ROIs of the bilateral occipital, parietal, frontal and temporal lobes and the cerebellum. Based on their involvement in ALD, we also defined ROIs in the total, genu, body and splenium of the corpus callosum and the internal capsule (anterior, posterior and rostral limbs combined). In the current paper, the corpus callosum ROIs were small binary ROIs based on the JHU atlas, as opposed to probabilistic tracts through the corpus callosum.^29^ In each ROI, we distinguished lesions and NAWM, and we determined the median value per quantitative measure. Within the bilateral (probabilistic) corticospinal tracts, the weighted means per measure were determined.

### Data processing

Image processing was performed as described previously.^29^ After preprocessing multi-shell DWI (denoising, geometrical distortion and motion correction), we analysed the single b0-b1000 shell data using DTIFIT to obtain FA, MD, RD and AD. We analysed the multi-shell data with the NODDI Watson model to obtain NDI. The outputs of both NODDI and BedpostX were used in subsequent analysis of MCR-DIMWI to obtain MWF_MCR-DIMWI_.^18,29^ METRICS data were analysed using spatial smoothness constraints to obtain MWF_METRICS._^29,36^ This analysis also yields an estimate of the short T2 relaxation time assigned to myelin water (MW-T2_METRICS_), which was extracted for exploratory analyses.

### Statistical analysis

Analyses were performed using SPSS version 28.0.1.1. Descriptive statistics are presented as means and standard deviations. First, to investigate the sensitivity of qMRI measures to lesions in cALD, values within lesions in the corpus callosum were compared to the whole corpus callosum of controls. This ROI was chosen for this analysis as it was the structure where the highest number of people with cALD (*n* = 9) had lesions. Next, variables that differentiated groups in the first comparison were compared between the NAWM of ALD participants with cALD, ALD participants without cALD and controls. Multivariate general linear models (GLMs) were used for analyses in male and female groups separately. FA and NDI (combined in the same GLM), MWF_MCR-DIMWI_ and MWF_METRICS_ in all ROIs were used as dependent variables. Disease status with three levels (cALD versus no cALD versus control) and scanner were used as fixed factors. Age was entered into the models as a covariate. If FA distinguished ALD participants from controls, AD and RD were investigated to identify the underlying cause of FA changes in more detail. Results were corrected for multiple comparisons according to Bonferroni. Estimated means, standard errors and *P*-values after correction are presented, unless indicated otherwise. An *α* < 0.05 was considered statistically significant. Effect sizes are expressed as partial eta-squared $\left(\eta_{p}^{2}\right)$. Effect sizes are small for $\eta_{p}^{2}$ = 0.01, medium for $\eta_{p}^{2}$ = 0.06 and large for 0.14 ≤ $\eta_{p}^{2}$ ≤ 1.0.^37^. Finally, as an exploratory analysis to further investigate microstructural abnormalities related to MWF_METRICS_, mean MW-T2_METRICS_ was compared between ALD participants and controls.

## Results

**Figure 2:**
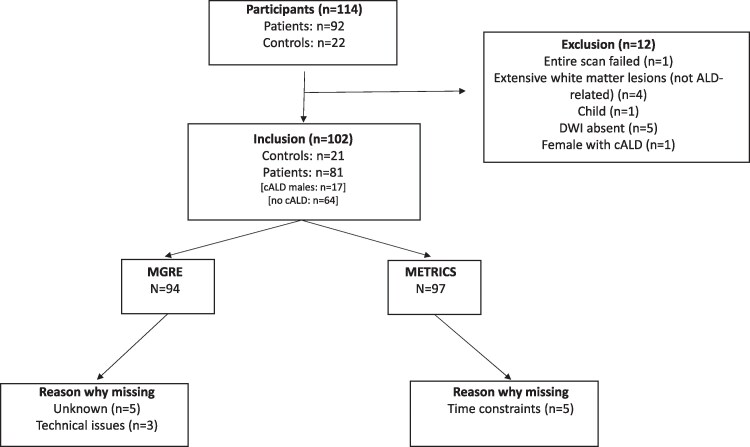
**Participant inclusion and exclusion.** DWI, diffusion-weighted imaging; cALD, cerebral ALD; MGRE, multi-echo gradient echo; METRICS, multi-echo T2 relaxation imaging with compressed sensing. Twelve individuals were excluded from the analyses for technical reasons, the presence of brain abnormalities not considered to be related to ALD or because they did not match the population inclusion criteria.

One hundred fourteen individuals were initially included in this study. Twelve participants (11%) were excluded (Fig. 2).

Notably, four participants (one male control and three males with ALD) had extensive white matter abnormalities that were considered not related to ALD but rather non-specific or of vascular origin. For five participants, multi-shell DWI was not collected or unusable, which precluded the definition of ROIs and tracts. One female with cALD was found to have a strongly skewed X-inactivation pattern. She was excluded from the study. A total of 81 ALD participants (69% males) and 21 controls (48% males) were included in the final analyses (Table 1). Seventeen males had MRI lesions consistent with cALD. Five of these males had undergone cell therapies that halted cALD progression. Although a gadolinium contrast agent was not recently administered in all cases, most males with cALD had stable lesions over (at least) a 1-year period and were therefore considered as having arrested or stable cALD. One male suffered from active cALD with contrast enhancement at the time of his scan. The location and extent of cALD lesions differed between subjects and were identified primarily in the corpus callosum, and in the periventricular white matter. The prevalence of lesions per anatomical structure is described in more detail in Supplementary Table 1. Within the ALD groups, 82% (*n* = 46) of males and 88% (*n* = 22) of females had signs and/or symptoms consistent with a myelopathy.

**Table 1:** Background characteristics

	Male patients (*n* = 56)	Female patients (*n* = 25)	Controls (*n* = 21)
Age, years (mean ± SD)	42 ± 16	53 ± 14	33 ± 12
Males (*n*, %)	NA	NA	10 (48%)
Scanner a/b/c	22/33/1	23/2/0	Male: 3/4/3 Female: 6/3/2
Myelopathy^a^ (*n*, %)	46 (82%)	22 (88%)	NA
Cerebral ALD (*n*, %)	17 (30%)	NA	NA
MGRE present (*n*, %)	52 (93%)	22 (88%)	20 (95%)
METRICS present (*n*, %)	52 (93%)	26 (100%)	19 (91%)

MGRE, multi-echo gradient echo; METRICS, multi-echo T2 relaxation imaging with compressed sensing.

^a^Myelopathy as illustrated by an expanded disease disability score >0. Scanner a is Philips Ingenia Elition X and scanner b and c are Philips Ingenia.

### qMRI measures in cALD lesions in the corpus callosum of males

FA, NDI, MWF_METRICS_ and MWF_MCR-DIMWI_ were significantly reduced within lesions in the corpus callosum in males with cALD (*n* = 9) compared to male controls (*n* = 10) (Table 2). AD and RD were significantly increased in these lesions compared to controls. Large effect sizes were observed for all measures.

**Table 2:** qMRI measures in lesions within the corpus callosum of male cALD patients and in male controls

	Estimated means (standard error)	Effect size $\eta_{p}^{2}$
FA		
Patient	0.309 (0.031)	0.820^a^
Control	0.602 (0.023)	
NDI		
Patient	0.267 (0.044)	0.823^a^
Control	0.693 (0.033)	
MWF_METRICS_		
Patient	0.054 (0.011)	0.812^a^
Control	0.145 (0.008)	
MWF_MCR-DIMWI_		
Patient	0.054 (0.007)	0.691^a^
Control	0.097 (0.005)	
AD (10^−5^ mm^2^/s)		
Patient	168.0 (9.1)	0.399^a^
Control	135.0 (6.9)	
RD (10^−5^ mm^2^/s)		
Patient	104.7 (9.4)	0.667^a^
Control	44.7 (7.1)	

A total of *n* = 9 (FA, NDI, AD, RD), *n* = 7 (MWF_METRICS_) and *n* = 8 (MWF_MCR-DIMWI_) male cALD patients with leukodystrophy lesions and *n* = 10 male controls were included in the analyses. Estimated means and standard errors are estimated using age and scanner as covariates and are corrected according to Bonferroni.

^a^ *P*-value < 0.05.

### qMRI measures in the NAWM

**Figure 3:**
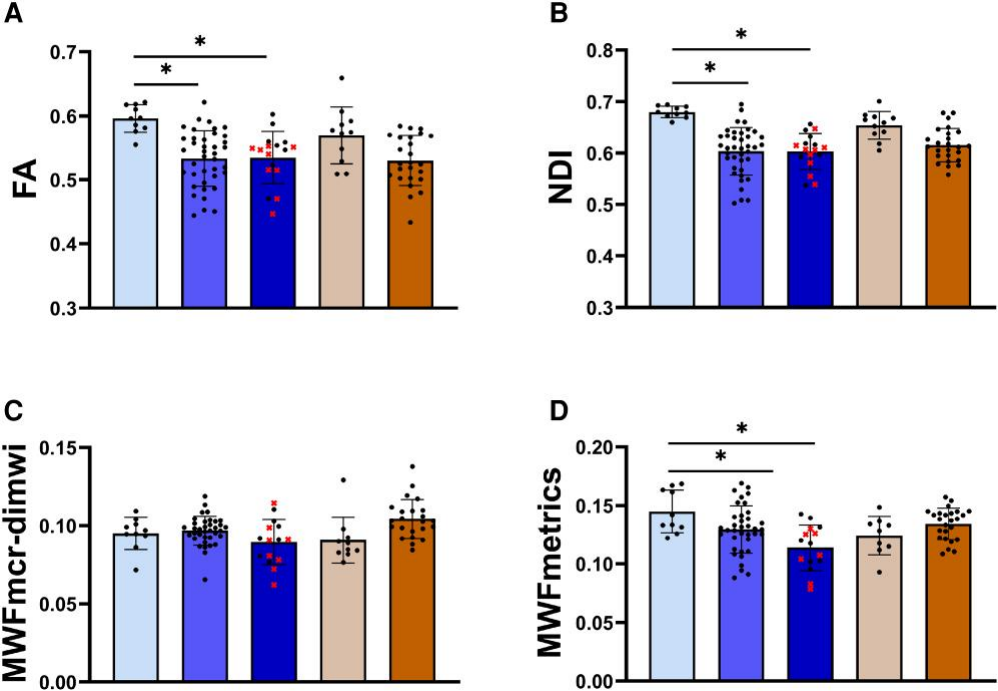
**FA, NDI and MWF in the NAWM of the corpus callosum.** From left to right: male controls (light blue, *n* = 10), males without cALD (medium blue, *n* = 39), males with cALD (dark blue, *n* = 17), female controls (beige, *n* = 11), females without cALD (brown, *n* = 25). Data points represent individual participants. Boxplots represent means and standard deviations. Red crosses in the cALD bars indicate participants with lesions in the corpus callosum (*n* = 9). For these participants, qMRI measures were determined in NAWM, which did not include lesion voxels. FA, NDI, and MWF are unitless fractions between 0 and 1. GLMs showed that FA (**A**), NDI (**B**) and MWF_METRICS_ (**D**) are significantly decreased in the NAWM of the corpus callosum in males with ALD compared to male controls. No significant differences were observed for MWF_MCR-DIMWI_ (**C**). In females, no significant differences were observed in this region. NAWM, normal-appearing white matter; FA, fractional anisotropy; NDI, neurite density index; MWF_MCR-DIMWI_, myelin-water fraction with diffusion-informed myelin-water imaging for the multi-echo gradient echo sequence; MWF_METRICS_, myelin-water fraction for the multi-echo T2 relaxation imaging with compressed sensing sequence. *Significant (*P* < 0.05) after Bonferroni correction for multiple testing corrected for age and for scanner.

A selection of the comparisons of FA, NDI, MWF_MCR-DIMWI_ and MWF_METRICS_ between the NAWM of ALD participants with cALD, the NAWM of ALD participants without cALD and controls are shown in Fig. 3 and in Table 3. The male cALD group includes both participants with lesions in the corpus callosum and participants with lesions elsewhere in the brain. Visually, there were no clear differences in qMRI measures between these two subgroups (red crosses for callosal lesions versus black dots for lesions elsewhere in the brain).

**Table 3:** FA, NDI, MWF_METRICS_ and MWF_MCR-DIMWI_ in the NAWM of male participants

	Splenium of corpus callosum			Corticospinal tracts		
	cALD	No cALD	Control	cALD	No cALD	Control
FA—participants (*n*)	17	39	10	17	39	10
FA—EM (SE)	0.522 (0.018)^a,b^	0.594 (0.013)	0.635 (0.018)	0.454 (0.008)^a^	0.465 (0.006)^a^	0.506 (0.008)
FA—$\eta_{p}^{2}$	0.306			0.284		
NDI—participants (*n*)	17	39	10	17	39	10
NDI—EM (SE)	0.619 (0.016)^a,b^	0.657 (0.012)^a^	0.725 (0.016)	0.670 (0.008)^a^	0.684 (0.006)^a^	0.737 (0.008)
NDI—$\eta_{p}^{2}$	0.289			0.430		
MWF_METRICS_—participants (*n*)	15	37	10	15	37	10
MWF_METRICS_—EM (SE)	0.118 (0.007)^a^	0.131 (0.005)	0.152 (0.007)	0.122 (0.006)^a^	0.132 (0.004)^a^	0.153 (0.006)
MWF_METRICS_—$\eta_{p}^{2}$	0.182			0.206		
MWF_MCR-DIMWI_—participants (*n*)	16	36	10	16	36	10
MWF_MCR-DIMWI_—EM (SE)	0.108 (0.009)	0.107 (0.007)	0.104 (0.009)	0.089 (0.009)	0.082 (0.007)	0.087 (0.009)
MWF_MCR-DIMWI_—$\eta_{p}^{2}$	0.003			0.012		

Results are shown for male patients with cALD, male patients without cALD (no cALD) and male controls in two representative regions. Measures represent estimated means (EM) and standard errors (SE) for normal-appearing white matter (NAWM) generated by GLMs. EM and SE are estimated with scanner and age as covariates and corrected for Bonferroni. FA, fractional anisotropy; NDI, neurite density index; MWF_METRICS_, myelin-water fraction obtained from METRICS (multi-echo T2 relaxation imaging with compressed sensing); MWF_MCR-DIMWI_, myelin-water fraction obtained with MCR-DIMWI (multi-compartment relaxometry diffusion-informed MWI).

^a^Significant difference (*P* < 0.05) between patient group and controls.

^b^Significant difference between cALD and no cALD.

Table 3 highlights the estimated means for two ROIs: the splenium of corpus callosum (often primarily affected in cALD), and the intracranial corticospinal tracts (often primarily affected in cALD and ALD spinal cord disease). All measures in all ROIs are shown in Supplementary Tables 2–5.

In the NAWM of males with cALD (*n* = 17), FA was significantly lower in 7 out of 10 ROIs compared to controls (*n* = 10) (Supplementary Table 2). In the NAWM of males without cALD (*n* = 39), FA was significantly reduced in 6 out of 10 ROIs. In four regions (splenium of the corpus callosum, parietal WM, temporal WM and cerebellum), FA was significantly lower in the males with cALD compared to the males without cALD. Effect sizes were large with the highest values found in the cerebellum ($\eta_{p}^{2}$ = 0.352) and splenium of corpus callosum ($\eta_{p}^{2}$ = 0.306).

In the NAWM of males with cALD (*n* = 17), NDI was significantly reduced in all ROIs compared to controls (*n* = 10) (Supplementary Table 3). Also in the NAWM of males without cALD (*n* = 39), NDI was significantly reduced in all ROIs. In two regions (splenium of the corpus callosum and cerebellum), NDI was significantly lower in the males with cALD compared to the males without cALD. The largest effect sizes were found in the internal capsule ($\eta_{p}^{2}$ = 0.345) and the corticospinal tracts ($\eta_{p}^{2}$ = 0.430).

In the NAWM of males with cALD (*n* = 15), MWF_METRICS_ was significantly decreased in 7 out of 10 ROIs (Supplementary Table 5). In the NAWM of males without cALD (*n* = 37), MWF_METRICS_ was significantly reduced in four ROIs. No significant differences were observed between males with and without cALD. Effect sizes were largest in the corpus callosum (genu: $\eta_{p}^{2}$ = 0.168; body: $\eta_{p}^{2}$ = 0.199; splenium: $\eta_{p}^{2}$ = 0.182), and in the corticospinal tracts ($\eta_{p}^{2}$ = 0.206).

Regarding MWF_MCR-DIMWI_, no significant differences were found between males with cALD (*n* = 16), males without cALD (*n* = 36) and male controls (*n* = 10) (Supplementary Table 4). Effect sizes were small.

Effect sizes for FA and NDI were larger than for MWF_METRICS_.

In the NAWM of females (all without cALD), no significant differences for FA (*n* = 25), NDI (*n* = 25) and MWF_METRICS_ (*n* = 25) were observed compared to controls (*n* = 11, *n* = 11, *n* = 9, respectively). In contrast to hypotheses, MWF_MCR-DIMWI_ (*n* = 23) was significantly increased compared to female controls (*n* = 10) in three ROIs.

### Axial and radial diffusivity in the NAWM

**Figure 4:**
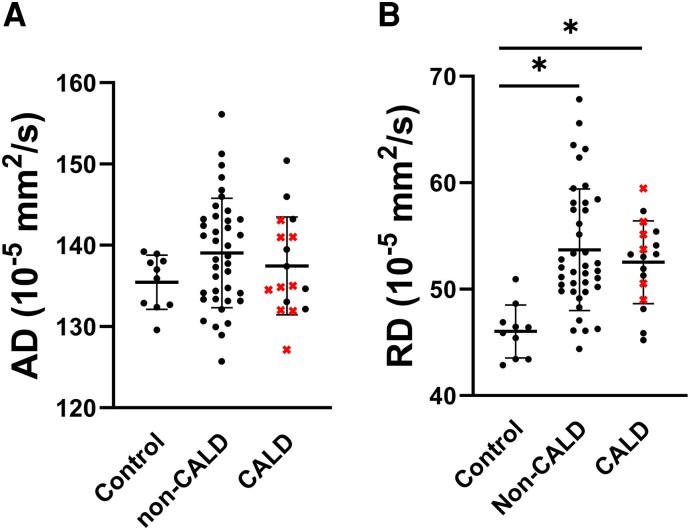
**AD and RD in the NAWM of the corpus callosum of males.** Boxplots represent mean and standard deviation of AD and RD in the NAWM of the corpus callosum. (**A**) GLMs showed that AD is not significantly different between the NAWM of males with cALD (*n* = 17), males without cALD (*n* = 39) and male controls (*n* = 10). (**B**) RD is significantly increased within the NAWM of both males with cALD and males without cALD. Crosses in the cALD group indicate participants with a lesion in the corpus callosum (*n* = 9). qMRI measures were determined in NAWM, which did not include lesion voxels. NAWM, normal-appearing white matter; AD, axial diffusivity; RD, radial diffusivity—both in units of 10^−5^ mm^2^/s. *Significant (*P* < 0.05) difference with control values after Bonferroni correction for multiple testing corrected for age and scanner.

Figure 4 shows results for AD and RD in the NAWM of the entire corpus callosum. The results for AD and RD in all ROIs in male participants are presented in Supplementary Table 6. Within the NAWM of males with cALD (*n* = 17), no significant differences in AD compared to controls (*n* = 10) were found in any of the ROIs. AD was increased in one ROI when comparing males without cALD (*n* = 39) to controls. RD was significantly increased in all 10 ROIs when comparing males with cALD to controls, and in seven ROIs when comparing males without cALD to controls. In the splenium of the corpus callosum and in the cerebellum, RD was higher in males with cALD compared to males without cALD.

### Myelin-water T2

As an exploratory analysis, the mean estimated T2 relaxation time of myelin water (MW-T2_METRICS_) of the METRICS protocol was evaluated. We hypothesized that the observed decrease in MWF_METRICS_ was mainly due to an increase of MW-T2_METRICS_ values of myelin water, such that it would shift beyond the 40 ms threshold and would be considered ‘intra-extracellular water’ instead of myelin water. Contrary to hypotheses, in lesions in the corpus callosum of males with cALD (*n* = 7), mean MW-T2_METRICS_ was lower (mean 11.9 ms, SD 4.5) than in controls (*n* = 10) (mean 18.0 ms, SD 2.8). Slightly lower values of MW-T2_METRICS_ were also observed in the NAWM in most ROIs of ALD males compared to controls (Supplementary Table 7).

## Discussion

This study examined DTI, NODDI and MWI measures in people with ALD. FA, AD, RD, NDI, MWF_MCR-DIMWI_ and MWF_METRICS_ differentiated lesions in the corpus callosum of males with cALD from male controls with large effect sizes, which confirms the sensitivity of these techniques to abnormal brain tissue. FA, NDI and MWF_METRICS_ were also significantly reduced in the NAWM of males, both with cALD and without cALD, in the majority of white matter ROIs. This shows that abnormalities are present at the tissue microstructure level in all parts of the brain white matter, suggesting cellular damage even when conventional MRI is normal. These abnormalities might functionally impair patients even when clear clinical manifestations are absent. Measures in males without cALD were generally closer to control values than measures in males with cALD, suggesting more severe tissue damage in the latter.

MWF_MCR-DIMWI_ did not differentiate NAWM of males with ALD from controls. By contrast, MWF_MCR-DIMWI_ was the only measure that differentiated the NAWM of females with ALD from female controls in a few ROIs, with higher values in patients than in controls. MWF values determined with both MWI techniques were previously found to be lower in patients with different types of leukodystrophy than in controls.^17,29^ The result of MWF_MCR-DIMWI_ in females is therefore unexpected. We assume that this qMRI measure is slightly variable between scanners and found a skewed distribution of females over the scanners, which, although we corrected for this in our analyses, may explain these results. Regarding the absence of differences in the other qMRI measures in females, the data suggest that the pathological process in women has a lower impact or no impact at all on the brain microstructure compared to males. This aligns with the fact that women generally do not develop leukodystrophy as a result of ALD.

When comparing quantitative measures in males, some of the largest effect sizes in the NAWM were observed for NDI. Neurite density (obtained with other diffusion models at ultrahigh field) has been associated with myelinated axons using optical and electron microscopy.^30^ Despite known limitations of the DTI model, such as the presence of crossing fibres, reductions of NDI went along with reductions in FA. In lesions, this was accompanied by increases in both RD and AD. In NAWM, RD was increased in almost all ROIs in males, both with and without cALD, whereas increases of AD were barely observed. RD is generally considered to reflect myelin integrity. These findings suggest pronounced abnormalities in both myelin and myelinated axons in males with ALD, even without cALD lesions on structural MRI. The abnormalities in RD and the relatively normal AD in the NAWM suggest that myelin damage may precede axonal damage in the earliest pathological stages of ALD.

cALD is characterized by disproportional and early involvement of the corpus callosum. The splenium of the corpus callosum, but also the genu and body, were found to be regions with some of the largest effect sizes for differences in qMRI measures within NAWM between males with ALD and controls, which suggests that these white matter structures are particularly affected by the biochemical defect of ALD. It is estimated that the lifetime risk of developing cALD for adult males with ALD is ∼ 20%.^38^ It may be possible that males with disproportionally low FA, NDI and MWF values in the corpus callosum are at higher risk of developing cALD. This hypothesis is supported by the observation that a few qMRI measures within the NAWM differed between males with cALD and males without cALD, mainly in the splenium and (unexpectedly) also in the cerebellum. Future prospective studies should investigate whether these measures can predict lesion development in an early stage. NDI differentiated males with ALD from controls with the largest effect sizes and may therefore be the most appropriate candidate measure for this purpose. Other ROIs that showed deviating qMRI measures with large effect sizes were the corticospinal tracts and internal capsules. These structures are also often affected by cALD.^2^ These findings are in line with previous studies that reported decreased FA in the cerebral corticospinal tracts in patients with ALD with normal conventional MRI.^27,39^ Possible relationships with the onset of clinical symptoms related to spinal cord disease and their use as biomarkers to monitor progression need to be established in future prospective studies.

MWI and NODDI are relevant in the context of ALD because of the pathological abnormalities in the axon-myelin units in brain lesions.^40-42^ The use of NODDI and MWI has been investigated in a number of neurological diseases, including white matter disorders.^43-48^ In MS, NODDI differentiated people with MS from controls and measures were correlated to disease severity.^49,50^ In ALD, a recent study showed that DTI measures were sensitive to early cALD lesions.^51^ The use of multi-shell DWI and MWI therefore has clinical potential, but their interpretation is challenging for several reasons. It should be noted that MWF is a measure of the amount of water between myelin sheaths, and not a direct measure of myelin density. When myelin sheaths become less compact due to pathology, the amount of water trapped between myelin sheaths (and thus perhaps MWF) increases. With further loosening of the sheaths, the T2 of myelin water will likely increase, and at some point, be considered intra-axonal or extracellular water, leading to a reduction of measured MWF. Relatively abnormal myelin could have pseudo-normal MWF, thus complicating straightforward MWF interpretation.^20^ To obtain a better understanding of the behaviour of myelin-water in ALD pathology, we investigated the MW-T2_METRICS_. A shorter MW-T2_METRICS_ was observed in lesions and in the NAWM of males with ALD compared to controls, suggesting that the reduced values of MWF_METRICS_ are not due to a shift of MW-T2_METRICS_ to intra- and extracellular water values. In the current ALD cohort, we hypothesize that the lower MW-T2_METRICS_ values may be due to increased VLCFA incorporation in myelin and perhaps other cellular membranes, thus changing its structure. These hypotheses should be further explored with MR experiments and pathological studies.

This study explored the use of NODDI and MWI in ALD. It included a large cohort of both male and female participants with and without cALD from different ages. Limitations are that the control group was smaller in size and younger than the ALD groups. Age was included as a covariate in all models to correct for this difference. Participants were scanned on one of three scanners and inter-scanner differences could have influenced results. MRI scanner was included as a fixed factor to account for these differences. Nevertheless, the variation in age and MRI scanner may have influenced results and should be further addressed in future studies. The males with cALD in this cohort had different types of lesions in different areas of the brain. Although gadolinium was not recently administered in all cases, most of them had stable lesions for more than 1 year and were therefore considered as having ‘arrested or stable’ cALD.^52^ Several participants had white matter lesions in the corpus callosum typical for ALD, whereas in others only the cerebellum or brainstem was (partially) affected. Differences between patients and controls may be more pronounced in a more homogeneous cohort. Related to this, the comparison of (sometimes small) lesions within the corpus callosum to the entire corpus callosum of controls may have biased results. We chose not to compare smaller regions because it would result in an even lower number of participants in the analysis. We used a relatively low spatial resolution for the qMRI methods in this study, with relatively large voxels of (2.5 mm).^3^ Although this is less optimal for characterizing very small lesions, the methods have a clinically feasible scanning time and a high signal-to-noise ratio. The spatial resolution is suitable for the investigation of NAWM ROIs.

NODDI and MWI are promising techniques to examine the brains of people with ALD *in vivo* and could be useful for monitoring. In this study, males with ALD with and without cALD lesions could be distinguished from controls, but no clear differences were observed for females. This indicates that damage of the white matter is on average more severe in males with ALD and can be observed before the occurrence of overt cALD lesions. The results in this study suggest that qMRI measures may be sensitive to the development of cALD in an early stage and may therefore have the potential to influence clinical decision-making. If these techniques are sensitive to change in individual patients, they could be used to predict leukodystrophy development, monitor the activity of lesions and to assess the effects of treatments. Future studies should investigate intra-participant longitudinal changes and correlations to clinical outcome measures.

## Supplementary Material

fcaf371_Supplementary_Data

## Data Availability

Data will be shared upon reasonable request to the authors. Analysis of MWF_MCR-DIMWI_ was done based on scripts in: https://github.com/kschan0214/mwi. Analysis of MWF_METRICS_ was done based on scripts that are available from: https://mriresearch.med.ubc.ca/news-projects/myelin-water-fraction/.
