# Supplementary material for: Myelin-water imaging and multi-shell diffusion-weighted imaging in adults with adrenoleukodystrophy
Source: Brain Commun. 2025 Sep 25;7(5):fcaf371. doi: 10.1093/braincomms/fcaf371 (PMC12514751; doi:10.1093/braincomms/fcaf371)
Supplement: fcaf371_Supplementary_Data [file fcaf371_supplementary_data.docx]

**Supplementary Table 1:** Lesion patterns in males with cALD (total 17 patients)

| **Region** | **Affected (n)** |
| --- | --- |
| Genu of corpus callosum | 4/17 |
| Body of corpus callosum | 1/17 |
| Splenium of corpus callosum | 8/17 |
| Internal capsule | 6/17 |
| Mesencephalon/cerebral peduncle | 6/17 |
| Brainstem (including pons, medulla oblongata) | 2/17 |
| Frontal white matter | 3/17 |
| Temporal/parietal white matter | 3/17 |
| Occipital white matter/optic radiation | 2/17 |
| Cerebellum/cerebellar peduncles | 3/17 |
| Periventricular white matter | 8/17 |

**Supplementary Table 2:** Fractional anisotropy in the normal-appearing white matter of patients and controls.

|  | **Male CALD (n=17)** | **Male no cALD (n=39)** | **Control (male) (n=10)** | **ηp2** | **Female non-CALD (n=25)** | **Control (female) (n=11)** | **ηp2** |
| --- | --- | --- | --- | --- | --- | --- | --- |
| **Frontal white matter** | 0.276 (0.005)* | 0.277 (0.004)* | 0.295 (0.005) | 0.129 | 0.283 (0.005) | 0.279 (0.006) | 0.014 |
| **Parietal white matter** | 0.282 (0.005)*^#^ | 0.296 (0.004)* | 0.316 (0.005) | 0.281 | 0.296 (0.006) | 0.301 (0.007) | 0.014 |
| **Occipital white matter** | 0.188 (0.007) | 0.203 (0.005) | 0.209 (0.007) | 0.093 | 0.197 (0.007) | 0.202 (0.007) | 0.008 |
| **Temporal white matter** | 0.265 (0.006)*^#^ | 0.282 (0.004) | 0.296 (0.006) | 0.227 | 0.281 (0.006) | 0.285 (0.006) | 0.010 |
| **Corpus callosum (genu)** | 0.492 (0.015) | 0.494 (0.011) | 0.533 (0.015) | 0.072 | 0.499 (0.018) | 0.488 (0.019) | 0.008 |
| **Corpus callosum (body)** | 0.531 (0.012) | 0.521 (0.009)* | 0.566 (0.012) | 0.129 | 0.550 (0.014) | 0.553 (0.015) | 0.001 |
| **Corpus callosum (splenium)** | 0.522 (0.018)*^#^ | 0.594 (0.013) | 0.635 (0.018) | 0.306 | 0.619 (0.015) | 0.641 (0.015) | 0.048 |
| **Capsula interna** | 0.543 (0.007)* | 0.547 (0.005)* | 0.585 (0.007) | 0.254 | 0.548 (0.009) | 0.564 (0.009) | 0.076 |
| **Cerebellum** | 0.248 (0.006)*^#^ | 0.271 (0.005)* | 0.294 (0.006) | 0.352 | 0.261 (0.006) | 0.272 (0.006) | 0.071 |
| **Corticospinal tracts** | 0.454 (0.008)* | 0.465 (0.006)* | 0.506 (0.008) | 0.284 | 0.465 (0.006) | 0.478 (0.006) | 0.101 |

Estimated means and standard errors are estimated using age and scanner as covariates, and corrected according to Bonferroni. * indicates a significant difference to controls (p-value <0.05). # indicates a significant difference between CALD and non-CALD patient groups.

**Supplementary Table 3:** Neurite density index in the normal-appearing white matter of patients and controls.

|  | **Male CALD (n=17)** | **Male non-CALD (n=39)** | **Male control (n=10)** | **ηp2** | **Female non-CALD (n=25)** | **Female control (n=11)** | **ηp2** |
| --- | --- | --- | --- | --- | --- | --- | --- |
| **Frontal white matter** | 0.514 (0.010)* | 0.521 (0.007)* | 0.566 (0.010) | 0.225 | 0.532 (0.010) | 0.529 (0.010) | 0.002 |
| **Parietal white matter** | 0.530 (0.010)* | 0.537 (0.007)* | 0.577 (0.010) | 0.183 | 0.547 (0.010) | 0.551 (0.010) | 0.004 |
| **Occipital white matter** | 0.497 (0.008)* | 0.500 (0.006)* | 0.527 (0.009) | 0.115 | 0.516 (0.007) | 0.517 (0.008) | 0.000 |
| **Temporal white matter** | 0.469 (0.008)* | 0.481 (0.006)* | 0.517 (0.008) | 0.269 | 0.486 (0.008) | 0.491 (0.008) | 0.007 |
| **Corpus callosum (genu)** | 0.541 (0.013)* | 0.554 (0.010)* | 0.617 (0.013) | 0.245 | 0.566 (0.014) | 0.578 (0.015) | 0.014 |
| **Corpus callosum (body)** | 0.605 (0.012)* | 0.603 (0.009)* | 0.668 (0.012) | 0.250 | 0.621 (0.011) | 0.637 (0.011) | 0.048 |
| **Corpus callosum (splenium)** | 0.619 (0.016)*^#^ | 0.657 (0.012)* | 0.725 (0.016) | 0.289 | 0.676 (0.015) | 0.702 (0.015) | 0.065 |
| **Capsula interna** | 0.615 (0.009)* | 0.627 (0.007)* | 0.683 (0.009) | 0.345 | 0.630 (0.011) | 0.653 (0.011) | 0.101 |
| **Cerebellum** | 0.577 (0.009)*^#^ | 0.603 (0.007)* | 0.647 (0.010) | 0.335 | 0.616 (0.008) | 0.616 (0.009) | 0.000 |
| **Corticospinal tracts** | 0.670 (0.008)* | 0.684 (0.006)* | 0.737 (0.008) | 0.430 | 0.699 (0.009) | 0.714 (0.009) | 0.063 |

Estimated means and standard errors estimated using age and scanner as covariates, and corrected according to Bonferroni. * indicates a significant difference to controls (p-value <0.05). # indicates a significant difference between CALD and non-CALD patient groups.

**Supplementary Table 4:** MCR-DIMWI myelin-water fraction (MWF_MCR-DIMWI_) in the normal-appearing white matter of patients and controls.

|  | **Male CALD (n=16)** | **Male non-CALD (n=36)** | **Male control (male) (n=10)** | **ηp2** | **Female non-CALD (n=23)** | **Female control (n=10)** | **ηp2** |
| --- | --- | --- | --- | --- | --- | --- | --- |
| **Frontal white matter** | 0.081 (0.007) | 0.072 (0.005) | 0.070 (0.007) | 0.038 | 0.074 (0.003)* | 0.062 (0.003) | 0.290 |
| **Parietal white matter** | 0.084 (0.007) | 0.074 (0.005) | 0.074 (0.007) | 0.038 | 0.072 (0.004)* | 0.061 (0.004) | 0.214 |
| **Occipital white matter** | 0.082 (0.009) | 0.068 (0.007) | 0.067 (0.009) | 0.047 | 0.070 (0.003) | 0.065 (0.004) | 0.069 |
| **Temporal white matter** | 0.085 (0.008) | 0.075 (0.006) | 0.075 (0.008) | 0.028 | 0.071 (0.003)* | 0.062 (0.004) | 0.161 |
| **Corpus callosum (genu)** | 0.099 (0.009) | 0.096 (0.006) | 0.095 (0.009) | 0.004 | 0.100 (0.009) | 0.082 (0.010) | 0.090 |
| **Corpus callosum (body)** | 0.097 (0.005) | 0.094 (0.004) | 0.094 (0.005) | 0.011 | 0.092 (0.004) | 0.082 (0.005) | 0.120 |
| **Corpus callosum (splenium)** | 0.108 (0.009) | 0.107 (0.007) | 0.104 (0.009) | 0.003 | 0.106 (0.006) | 0.094 (0.006) | 0.081 |
| **Capsula interna** | 0.100 (0.009) | 0.094 (0.007) | 0.094 (0.009) | 0.011 | 0.096 (0.005) | 0.088 (0.005) | 0.062 |
| **Cerebellum** | 0.058 (0.009) | 0.048 (0.006) | 0.047 (0.009) | 0.025 | 0.054 (0.003) | 0.048 (0.003) | 0.074 |
| **Corticospinal tracts** | 0.089 (0.009) | 0.082 (0.007) | 0.087 (0.009) | 0.012 | 0.085 (0.005) | 0.080 (0.006) | 0.020 |

Estimated means and standard errors estimated using age and scanner as covariates, and corrected according to Bonferroni. * indicates a significant difference to controls (p-value <0.05). # indicates a significant difference between CALD and non-CALD patient groups.

**Supplementary Table 5:** Metrics myelin-water fraction (MWF_METRICS)_ in the normal-appearing white matter of patients and controls.

|  | **Male patients CALD (n=15)** | **Male non-CALD (n=37)** | **Male control (n=10)** | **ηp2** | **Female patients non-CALD (n=25)** | **Female control (n=9)** | **ηp2** |
| --- | --- | --- | --- | --- | --- | --- | --- |
| **Frontal white matter** | 0.094 (0.004)* | 0.098 (0.003) | 0.109 (0.004) | 0.129 | 0.102 (0.005) | 0.095 (0.006) | 0.057 |
| **Parietal white matter** | 0.102 (0.004) | 0.107 (0.003) | 0.110 (0.004) | 0.036 | 0.114 (0.005) | 0.106 (0.005) | 0.069 |
| **Occipital white matter** | 0.098 (0.004) | 0.107 (0.003) | 0.106 (0.004) | 0.074 | 0.112 (0.006) | 0.099 (0.007) | 0.121 |
| **Temporal white matter** | 0.087 (0.004) | 0.093 (0.003) | 0.097 (0.004) | 0.070 | 0.098 (0.005) | 0.089 (0.005) | 0.088 |
| **Corpus callosum (genu)** | 0.114 (0.007)* | 0.121 (0.005)* | 0.145 (0.007) | 0.168 | 0.124 (0.007) | 0.117 (0.008) | 0.028 |
| **Corpus callosum (body)** | 0.120 (0.007)* | 0.128 (0.005)* | 0.152 (0.006) | 0.199 | 0.131 (0.007) | 0.124 (0.007) | 0.030 |
| **Corpus callosum (splenium)** | 0.118 (0.007)* | 0.131 (0.005) | 0.152 (0.007) | 0.182 | 0.151 (0.007) | 0.145 (0.007) | 0.022 |
| **Capsula interna** | 0.116 (0.006)* | 0.125 (0.004)* | 0.143 (0.006) | 0.191 | 0.121 (0.007) | 0.122 (0.007) | 0.001 |
| **Cerebellum** | 0.059 (0.005)* | 0.069 (0.004) | 0.083 (0.005) | 0.172 | 0.073 (0.006) | 0.065 (0.006) | 0.068 |
| **Corticospinal tracts** | 0.122 (0.006)* | 0.132 (0.004)* | 0.153 (0.006) | 0.206 | 0.143 (0.008) | 0.142 (0.008) | 0.001 |

Estimated means and standard errors estimated using age and scanner as covariates, and corrected according to Bonferroni. * indicates a significant difference to controls (p-value <0.05). # indicates a significant difference between CALD and non-CALD patient groups.

**Supplementary Table 6**: Axial and radial diffusivity in the normal-appearing white matter of male participants.

|  | **AD** | | | | **RD** | | | |
| --- | --- | --- | --- | --- | --- | --- | --- | --- |
|  | **Male CALD (n=17)** | **Male non-CALD (n=39)** | **Control (n=10)** | **ηp2** | **Male CALD (n=17)** | **Male non-CALD (n=39)** | **Control (n=10)** | **ηp2** |
| **Frontal white matter** | 101.4 (1.3) | 100.5 (1.0) | 97.6 (1.3) | 0.075 | 64.1 (1.1)* | 63.7 (0.8)* | 60.0 (1.1) | 0.125 |
| **Parietal white matter** | 101.2 (1.3) | 101.5 (1.0) | 98.7 (1.3) | 0.048 | 63.4 (1.2)* | 62.6 (0.8)* | 59.1 (1.1) | 0.129 |
| **Occipital white matter** | 101.3 (1.1) | 100.5 (0.8) | 99.3 (1.2) | 0.029 | 71.0 (1.1)* | 69.1 (0.8) | 67.0 (1.1) | 0.120 |
| **Temporal white matter** | 103.3 (0.8) | 103.9 (0.6)* | 101.5 (0.8) | 0.098 | 67.5 (0.8)* | 66.3 (0.6)* | 63.1 (0.8) | 0.221 |
| **Corpus callosum (genu)** | 131.2 (2.3) | 131.4 (1.7) | 127.2 (2.4) | 0.036 | 56.1 (1.6)* | 55.8 (1.2)* | 50.7 (1.6) | 0.114 |
| **Corpus callosum (body)** | 134.8 (2.4) | 132.5 (1.8) | 130.9 (2.5) | 0.026 | 52.4 (1.4)* | 53.3 (1.1)* | 47.3 (1.5) | 0.160 |
| **Corpus callosum (splenium)** | 147.8 (2.2) | 151 (1.6) | 146.8 (2.2) | 0.060 | 56.7 (1.9)*# | 51.0 (1.4) | 45.3 (1.9) | 0.265 |
| **Capsula interna** | 125.9 (1.2) | 126 (0.9) | 123.7 (1.2) | 0.039 | 49.1 (0.8)* | 48.9 (0.6)* | 44.0 (0.8) | 0.336 |
| **Cerebellum** | 101.4 (2.0) | 97.1 (1.5) | 97.0 (2.1) | 0.084 | 65.0 (1.8)*# | 59.8 (1.3) | 58.2 (1.8) | 0.165 |
| **Corticospinal tracts** | 131.1 (1.5) | 129 (1.1) | 127.2 (1.6) | 0.062 | 65.4 (1.6)* | 62.9 (1.2)* | 57.1 (1.7) | 0.190 |

Estimated means and standard errors estimated using age and scanner as covariates, and corrected according to Bonferroni. * indicates a significant difference to controls (p-value <0.05). # indicates a significant difference between CALD and non-CALD patient groups.

AD and RD are both in units of 10^-5^ mm²/s.

**Supplementary Table 7***:* Mean myelin-water T2 (MW-T2_METRICS_) in normal-appearing white matter (NAWM) in male participants

|  | **CALD (male) (n=15)** | **Non-CALD (male) (n=37)** | **Control (male) (n=10)** |
| --- | --- | --- | --- |
| **Frontal white matter** | 18.2 (2.3) | 17.1 (1.5) | 19.0 (2.2) |
| **Parietal white matter** | 16.7 (1.8) | 16.4 (1.2) | 18.6 (1.0) |
| **Occipital white matter** | 16.7 (1.3) | 16.7 (1.2) | 18.2 (0.9) |
| **Temporal white matter** | 17.8 (1.5) | 17.6 (1.4) | 20.1 (1.4) |
| **Corpus callosum (genu)** | 19.0 (4.9) | 18.5 (3.6) | 19.0 (4.3) |
| **Corpus callosum (body)** | 18.6 (4.6) | 17.8 (3.2) | 18.5 (3.0) |
| **Corpus callosum (splenium)** | 17.2 (4.1) | 15.1 (2.3) | 16.5 (1.9) |
| **Capsula interna** | 17.4 (3.4) | 16.5 (2.6) | 17.9 (2.1) |
| **Cerebellum** | 15.9 (3.1) | 16.0 (2.9) | 20.0 (1.8) |
| **Corticospinal tracts** | 17.0 (3.0) | 16.6 (2.3) | 18.7 (1.7) |

Calculated based on METRICS protocol. Presented as mean and (standard deviation). Measures are presented in milliseconds.
